# Longterm follow-up in European respiratory health studies – patterns and implications

**DOI:** 10.1186/1471-2466-14-63

**Published:** 2014-04-16

**Authors:** Ane Johannessen, Giuseppe Verlato, Bryndis Benediktsdottir, Bertil Forsberg, Karl Franklin, Thorarinn Gislason, Mathias Holm, Christer Janson, Rain Jögi, Eva Lindberg, Ferenc Macsali, Ernst Omenaas, Francisco Gomez Real, Eirunn Waatevik Saure, Vivi Schlünssen, Torben Sigsgaard, Trude Duelien Skorge, Cecilie Svanes, Kjell Torén, Marie Waatevik, Roy Miodini Nilsen, Roberto de Marco

**Affiliations:** 1 Centre for Clinical Research, Haukeland University Hospital, Bergen 5021, Norway; 2 Sezione di Epidemiologia & Statistica Medica, Università di Verona, Verona, Italy; 3 Department of Allergy, Respiratory Medicine and Sleep, Landspitali University Hospital, Reykjavik, Iceland; 4 Division of Occupational and Environmental Medicine, Department of Public Health and Clinical Medicine, Umeå University, Umeå, Sweden; 5 Surgical and Perioperative Sciences, Umeå University, Umeå, Sweden; 6 Department of Occupational and Environmental Medicine, Sahlgrenska University Hospital, Gothenburg, Sweden; 7 Department of Medical Sciences: Respiratory Medicine and Allergology, Uppsala University, Uppsala, Sweden; 8 Lung Clinic, University of Tartu, Tartu, Estonia; 9 Department of Obstetrics and Gynecology, Haukeland University Hospital, Bergen, Norway; 10 Institute of Medicine, Bergen University, Bergen, Norway; 11 Department of Public Health, Section for Environment, Work and Health, Aarhus University, Aarhus, Denmark; 12 Department of Occupational Medicine, Aarhus University Hospital, Aarhus, Denmark; 13 Department of Occupational Medicine, Haukeland University Hospital, Bergen, Norway; 14 Section of Occupational and Environmental Medicine, University of Gothenburg, Gothenburg, Sweden

## Abstract

**Background:**

Selection bias is a systematic error in epidemiologic studies that may seriously distort true measures of associations between exposure and disease. Observational studies are highly susceptible to selection bias, and researchers should therefore always examine to what extent selection bias may be present in their material and what characterizes the bias in their material. In the present study we examined long-term participation and consequences of loss to follow-up in the studies Respiratory Health in Northern Europe (RHINE), Italian centers of European Community Respiratory Health Survey (I-ECRHS), and the Italian Study on Asthma in Young Adults (ISAYA).

**Methods:**

Logistic regression identified predictors for follow-up participation. Baseline prevalence of 9 respiratory symptoms (asthma attack, asthma medication, combined variable with asthma attack and/or asthma medication, wheeze, rhinitis, wheeze with dyspnea, wheeze without cold, waking with chest tightness, waking with dyspnea) and 9 exposure-outcome associations (predictors sex, age and smoking; outcomes wheeze, asthma and rhinitis) were compared between all baseline participants and long-term participants. Bias was measured as ratios of relative frequencies and ratios of odds ratios (ROR).

**Results:**

Follow-up response rates after 10 years were 75% in RHINE, 64% in I-ECRHS and 53% in ISAYA. After 20 years of follow-up, response was 53% in RHINE and 49% in I-ECRHS. Female sex predicted long-term participation (in RHINE OR (95% CI) 1.30(1.22, 1.38); in I-ECRHS 1.29 (1.11, 1.50); and in ISAYA 1.42 (1.25, 1.61)), as did increasing age. Baseline prevalence of respiratory symptoms were lower among long-term participants (relative deviations compared to total baseline population 0-15% (RHINE), 0-48% (I-ECRHS), 3-20% (ISAYA)), except rhinitis which had a slightly higher prevalence. Most exposure-outcome associations did not differ between long-term participants and all baseline participants, except lower OR for rhinitis among ISAYA long-term participating smokers (relative deviation 17% (smokers) and 44% (10–20 pack years)).

**Conclusions:**

We found comparable patterns of long-term participation and loss to follow-up in RHINE, I-ECRHS and ISAYA. Baseline prevalence estimates for long-term participants were slightly lower than for the total baseline population, while exposure-outcome associations were mainly unchanged by loss to follow-up.

## Background

Large prospective population-based studies provide important evidence for public health interventions aiming at early disease prevention and treatment [1,2]. However, in order to draw valid scientific conclusions, data must be collected in a way that minimizes systematic errors [3]. Failing to avoid such errors in data collection could compromise the internal validity of exposure-outcome associations, leading to biased effect estimates and erroneous conclusions [1,4,5].

In a population-based follow-up study data is collected repeatedly within the same cohort of study participants. Inevitably, this study design is vulnerable to loss to follow-up. If loss to follow-up is greater in some exposure groups than others, it can affect prevalence estimates and in some cases also exposure-outcome association estimates [6-9]. Thus, an evaluation of non-response and loss to follow-up is essential in order to determine the validity and scientific potential of population-based epidemiological studies.

In 1989, the largest European longitudinal study within the field of respiratory health was launched; the European Community Respiratory Health Survey (ECRHS) [10]. In relation to this study, Northern European countries initiated a study with postal questionnaires expanding the baseline ECRHS population to include representative populations in Iceland, Denmark, Sweden, Norway and Estonia: the Respiratory Health in Northern Europe (RHINE) study [11]. Also in Southern Europe study centers were involved in both the ECRHS as well as formed separate studies in relation with ECRHS. Italian Study on Asthma in Young Adults (ISAYA) is one such study [12].

The aim of the present paper was to examine long-term participation and consequences of loss to follow-up in Northern European and Italian study centers. We aimed to identify predictors for long-term participation, and to quantify bias in selected respiratory outcomes and exposure-outcome associations.

## Methods

### Study population

**Figure 1:**
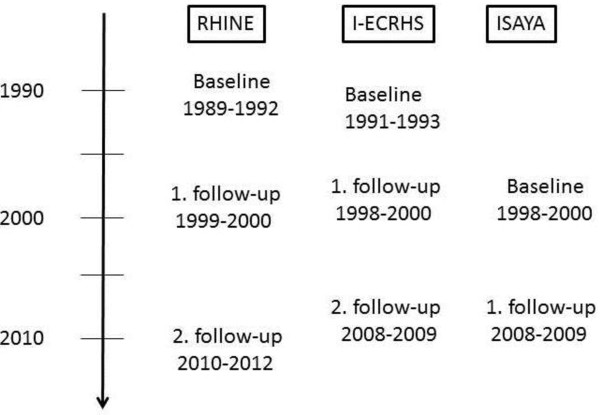
Flow chart of the RHINE, I-ECRHS and ISAYA studies.

The overall aims of RHINE, I-ECRHS and ISAYA are to identify incidence, prevalence and risk factors for respiratory diseases such as asthma and chronic obstructive pulmonary disease, and symptoms related to such diseases. RHINE is a large Northern European prospective cohort study initiated in 1989–1992, with follow-ups in 1999–2000 and in 2010–2012 (Figure 1). Participating centers in RHINE are Reykjavik (Iceland), Bergen (Norway), Umeå, Uppsala and Gothenburg (Sweden), Aarhus (Denmark) and Tartu (Estonia) [11,13].

The Italian centers included in the ECRHS are Verona, Pavia and Turin. They were all included in the ECRHS in 1991–93 with a follow-up examination in 1998–2000 [14]. Verona also completed a second follow-up in 2008–2009 (Figure 1).

ISAYA was initially conducted in 1998–2000 and comprised nine study centers [12]. Of these, two centers, Verona and Sassari, participated in a follow-up study in 2008–2009 (Figure 1).

We examined data from the total baseline populations in each of the three studies, and compared them with baseline data for 10-yrs follow-up populations (subjects who participated both at baseline and first follow-up in the studies) and with baseline data for 20-yrs follow-up populations (subjects who participated both at baseline and both follow-up examinations in RHINE and I-ECRHS). We also examined data on associations of smoking with selected outcomes for all three studies in 1998–2000 (first follow-up study for RHINE and I-ECRHS, and baseline for ISAYA) and compared them with the same data for 10-yrs follow-up populations. The selection of 1998–2000 data for these exposure-outcome analyses was due to missing smoking information at baseline for RHINE and I-ECRHS.

In all studies, informed consent was obtained from each participant prior to each stage, and the studies were approved by regional committees of medical research ethics according to national legislations. For exact names on the regional ethics committees in each study centre, please see information in the online supplement.

### Selected outcomes and exposures

The data used for the present study was collected through questionnaires with the same questions in all three studies. In RHINE, the data was collected through self-administered questionnaires, while in the Italian studies the data was collected partly through self-administered questionnaires (66% in I-ECRHS and 72% in ISAYA) and partly by telephone interviews (44% in I-ECRHS and 28% in ISAYA) [14,15]. Main outcomes for the present study were wheeze, asthma and rhinitis (see online supplement for exact question wording). In the online supplement, we also present baseline prevalence of other respiratory symptoms: wheeze with dyspnea, wheeze without cold, waking with chest tightness, waking with dyspnea, asthma attack last 12 months, and current asthma medication.

Selected exposure variables were sex and age at baseline, as well as study center. In addition, we inspected associations of wheeze, asthma, rhinitis with smoking exposure. Smoking variables were self-reported never/ex/current smoker, and smokers defined as <10 pack years, 10–20 pack years and ≥20 pack years, with one pack year being defined as having smoked 20 cigarettes a day for one year.

### Statistical methods

All analyses were performed using Stata/SE version 12.1 (StataCorp, Texas, USA) software for Windows. Logistic regression analyses were performed to estimate associations of age, sex and study center with long-term participation, using a binary indicator of participation in follow-up (0 = no, 1 = yes) as dependent variable.

When examining if prevalence and association estimates differed between all baseline and long-term participants, we followed methods used by among others Nilsen et al., using baseline data as the reference [8,16-19]. The methodology used by Nilsen et al. is described in detail in the remainder of this section, applying it to the focus of interest in the present study. We estimated baseline prevalence (with 95% confidence intervals) of all respiratory outcomes for all baseline participants, for those who participated at baseline and first follow-up (10-yrs follow-up), and for those who participated at baseline and both follow-ups (20-yrs follow-up). We assessed ratios of baseline prevalence of long-term participants over all baseline participants, in order to examine potential bias in prevalence between these various populations. The 9 selected exposure-outcome associations were investigated through logistic regression analyses, and ratios of baseline ORs among the various forms of long-term participants over all baseline participants were calculated [8,16,18]. In both ratios of prevalence estimates and ratios of ORs, a ratio below 1 indicates under-estimation in the subsample compared to the total baseline population (long-term participants have a lower prevalence or weaker exposure-outcome association than all baseline participants), while a ratio above 1 indicates an over-estimation (long-term participants have a higher prevalence or stronger exposure-outcome association). For ratios of ORs, this interpretation is reversed if the exposure has a protective effect on the outcome.

For both ratios of prevalence estimates and ratios of ORs, we computed 95% confidence intervals to assess the uncertainty of the ratio through bootstrapping [20]. For each of the studies, we identified long-term participants (n) and the remainder of the baseline population (m) in the total baseline population data file (m + n). We performed 2000 random re-samplings from the total baseline population, and created 2000 alternative data sets with size m + n. For each sample, we computed the ratios of long-term participants (n) over all baseline participants (m + n). By extracting the 2.5 percentile and the 97.5 percentile from these 2000 ratio estimates, we retrieved the 95% confidence interval.

## Results

### Response rates

In RHINE, the baseline study in 1989–1992 comprised 21 659 subjects aged 20–44 yrs (Table 1). In the first follow-up in 1999–2000, 75% answered a new questionnaire. In the second follow-up in 2010-12, response rate among those who had participated in the previous two stages was 53%.

**Table 1:** **Response rates by study centres at baseline and follow-up stages**^**a**^

***Study center***	***Baseline***	***10-yrs follow-up***	***20-yrs follow-up***
RHINE			
Aarhus	3614 (85%)	2 589 (72%)	1959 (54%)
Bergen	3449 (82%)	2 506 (73%)	1833 (53%)
Gothenburg	2861 (83%)	2 175 (76%)	1496 (52%)
Reykjavik	2899 (84%)	1 967 (68%)	1572 (54%)
Tartu	2449 (85%)	1 705 (70%)	1066 (44%)
Umea	3273 (92%)	2 621 (80%)	1745 (53%)
Uppsala	3114 (89%)	2 543 (82%)	1770 (57%)
TOTAL	21 659 (86%)	16 106 (75%)	11 441 (53%)
Italian ECRHS			
Verona	2711 (92%)	1737 (64%)	1338 (49%)
Pavia	816 (86%)	701 (86%)	-
Turin	2502 (84%)	1443 (58%)	-
TOTAL	6 029 (88%)	3 881 (64%)	1338 (49%)^b^
ISAYA			
Verona	2158 (74%)	1421 (66%)	-
Sassari	2053 (70%)	810 (39%)	-
TOTAL	4 211 (72%)	2 231 (53%)	-

^a^Subjects who have answered one or more of the following questions at each of the relevant study stages: asthma, hay fever, wheeze. Baseline: 1989–92 in RHINE, 1991–93 in Italian ECRHS, 1998–2000 in ISAYA. 10-yrs follow-up: 1999–2000 in RHINE, 1998-2000 in Italian ECRHS, 2008–09 in ISAYA. 20-yrs follow-up: 2010–12 in RHINE, 2008–09 in Italian ECRHS.

Response rates are calculated based on those invited to baseline for baseline populations, and based on those participating in baseline for follow-up populations.

^b^Response rate for 20-yrs follow-up in Italian ECRHS is calculated based on original response rate in Verona.

In I-ECRHS centers, the baseline study in 1991–93 comprised 6 029 subjects aged 20–45 yrs. In the first follow-up in 1998–2000, response rate was 64%, and from the Verona study center 49% participated also in the second follow-up in 2008–09.

In ISAYA, 4 211 subjects aged 20–45 yrs participated at baseline in 1998–2000. At follow-up 10 yrs later, 53% participated. The initial response rates at baseline were high across all centres, varying from 70% in Sassari (Italy) to 92% in Umeå (Sweden) and Verona (Italy) (Table 1, [12,21]). Ten years later, response rates varied from 39% in Sassari (Italy) to 86% in Pavia (Italy). When looking at participants 20 years after baseline, response rates varied from 44% in Tartu (Estonia) to 57% in Uppsala (Sweden).

### Determinants of participation

Table 2 presents associations of age, sex and study center, with 10-yrs and 20-yrs follow-up participation, respectively. OR for long-term participation increased with increasing age, especially in RHINE and I-ECRHS. Women were more often long-term participants than men in all three studies. The propensity to participate varied significantly across centers: the OR for long-term participation in RHINE was especially high in Umea and Uppsala for 10-yrs follow-up, and in Aarhus and Uppsala for 20-yrs follow-up. In I-ECRHS and in ISAYA, Pavia and Verona had the highest ORs for long-term participation, respectively.

**Table 2:** **Predictors for long-term participation in RHINE, Italian ECRHS and ISAYA, odds ratios with 95**% **confidence intervals**

***Study centre***		***10-yrs follow-up***	***20-yrs follow-up***
RHINE	20-25 yrs	Ref	Ref
	25-30 yrs	1.18 (1.08, 1.29)	1.15 (1.06, 1.24)
	30-35 yrs	1.30 (1.19, 1.42)	1.31 (1.20, 1.42)
	35-40 yrs	1.33 (1.21, 1.46)	1.40 (1.29, 1.52)
	40-44 yrs	1.55 (1.40, 1.72)	1.59 (1.45, 1.73)
	Men	Ref	Ref
	Women	1.30 (1.22, 1.38)	1.28 (1.21, 1.35)
	Aarhus	1.22 (1.10, 1.36)	1.50 (1.35, 1.66)
	Bergen	1.29 (1.16, 1.44)	1.44 (1.30, 1.60)
	Gothenburg	1.52 (1.35, 1.70)	1.37 (1.22, 1.52)
	Reykjavik	Ref	1.47 (1.31, 1.64)
	Tartu	1.14 (1.01, 1.28)	Ref
	Umea	1.93 (1.72, 2.17)	1.42 (1.28, 1.58)
	Uppsala	2.14 (1.90, 2.41)	1.65 (1.48, 1.83)
Italian	20-25 yrs	Ref	Ref
ECRHS	25-30 yrs	0.91 (0.77, 1.07)	0.96 (0.76, 1.22)
	30-35 yrs	1.07 (0.90, 1.27)	1.27 (1.00, 1.63)
	35-40 yrs	1.29 (1.08, 1.54)	1.50 (1.17, 1.92)
	40-44 yrs	1.50 (1.26, 1.80)	1.63 (1.26, 2.09)
	Men	Ref	Ref
	Women	1.18 (1.06, 1.31)	1.29 (1.11, 1.50)
	Verona	1.33 (1.19, 1.49)	-
	Pavia	4.48 (3.62, 5.55)	-
	Turin	Ref	-
ISAYA	20-25 yrs	Ref	-
	25-30 yrs	1.08 (0.89, 1.32)	-
	30-35 yrs	1.19 (0.97, 1.45)	-
	35-40 yrs	1.29 (1.05, 1.60)	-
	40-44 yrs	1.19 (0.96, 1.47)	-
	Men	Ref	-
	Women	1.42 (1.25, 1.61)	-
	Verona	2.93 (2.59, 3.33)	-
	Sassari	Ref	-

### Baseline prevalence of respiratory symptoms

Prevalence estimates of wheeze, asthma and rhinitis at baseline are shown in Table 3 and in Additional file 1: e-Table S1 in the online supplement for each study center separately. Baseline prevalence of several other respiratory symptoms is presented in the online supplement (Additional file 1: e-Table S2). In RHINE, prevalence of baseline wheeze last 12 months, wheeze with dyspnea, wheeze without cold, waking with chest tightness and waking with dyspnea were significantly lower in the long-term participants compared to the total baseline population, while the prevalence of rhinitis was higher. Waking with dyspnea had a relative deviation of 15% between the 20-yrs follow-up participants and the total baseline population, while all other symptoms differed by <10% between the long-term population and the total baseline population.

**Table 3:** **Prevalence (with 95**% **confidence intervals) of respiratory symptoms at baseline in total baseline population, population at 10-yrs follow-up and population at 20-yrs follow-up, and differences between these groups given as ratios of baseline symptom prevalence (with bootstrapped 95**% **confidence intervals) in population at 10-yrs follow-up over total baseline population, and in population at 20-yrs follow-up over total baseline population**^**a**^

		***Baseline population (95%CI)***	***10-yrs follow-up population (95%CI)***	***Ratio of prevalences (95%CI)***^***b***^	***20-yrs follow-up population (95%CI)***	***Ratio of prevalences (95%CI)***^***b***^
Wheeze	RHINE	22.2% (20.4, 21.6)	21.5% (19.9, 21.3)	**0.97 (0.95, 0.99)**	20.5% (19.0, 20.6)	**0.92 (0.90, 0.95)**
	Italian ECRHS	10.0% (9.0, 10.5)	10.2% (8.9, 10.8)	1.02 (0.96, 1.07)	10.1% (8.0, 11.2)	1.01 (0.87, 1.14)
	ISAYA	15.1% (12.4, 14.6)	13.7% (11.1, 13.8)	**0.91 (0.84, 0.98)**	-	-
Asthma	RHINE	4.7% (4.3, 5.0)	4.8% (4.4, 5.2)	1.02 (0.99, 1.05)	4.6% (4.3, 5.1)	0.98 (0.92, 1.03)
	Italian ECRHS	4.2% (3.6, 4.6)	4.2% (3.5, 4.7)	1.00 (0.91, 1.09)	3.8% (2.5, 4.5)	0.90 (0.67, 1.10)
	ISAYA	5.3% (4.4, 5.8)	5.0% (3.8, 5.6)	0.94 (0.83, 1.07)	-	-
Rhinitis	RHINE	19.3% (19.3, 20.5)	19.8% (19.7, 21.1)	**1.03 (1.01, 1.04)**	19.9% (19.9, 21.5)	**1.03 (1.00, 1.06)**
	Italian ECRHS	15.9% (14.8, 16.6)	15.8% (14.4, 16.7)	0.99 (0.95, 1.03)	18.2% (15.8, 20.0)	**1.14 (1.02, 1.26)**
	ISAYA	20.7% (19.3, 21.8)	21.1% (19.3, 22.7)	1.02 (0.96, 1.08)	-	-

^a^Total baseline population: participants in 1989–92 in RHINE, 1991–93 in Italian ECRHS, 1998–2000 in ISAYA. 10-yrs follow up population: participants in 1999-2000 in RHINE and 1998–2000 in Italian ECRHS, 2008–09 in ISAYA. 20-yrs follow-up population: participants in 2010-12 in RHINE, 2008–09 in Italian ECRHS.

^b^Corrected for inter-dependency between long-term participants and total baseline participants, by using a non-parametric bootstrap method. Significant differences between long-term participants and all baseline participants are marked in bold font.

In I-ECRHS, the 10-yrs follow-up population and the total baseline population did not differ in baseline prevalence of any of the respiratory outcomes. Regarding 20-yrs follow-up, however, baseline prevalence of rhinitis was higher compared to the corresponding estimate in the total baseline population (relative deviation 14%), while wheeze with dyspnea and waking with dyspnea was lower (relative deviations 48% and 23%, respectively). In ISAYA, baseline prevalence of wheeze last 12 months, wheeze with dyspnea and waking with chest tightness was lower in the 10-yrs follow-up population compared to the total baseline population (relative deviations 9%, 20% and 11%, respectively).

A closer look at the study centers (Additional file 1: e-Table S1) shows more heterogeneous study centers in RHINE than in I-ECRHS and ISAYA. In Reykjavik, baseline asthma and rhinitis was higher in long-term participants compared to total baseline population, the same was true for rhinitis in Tartu and Umea. Aarhus, Bergen, Gothenburg and Tartu had lower baseline prevalence of wheeze among long-term participants than among all baseline participants, for Aarhus this was also the case with asthma.

### Associations of age and sex with respiratory outcomes

Table 4 shows ORs for age (5-year-intervals) and female sex with regard to baseline wheeze, asthma and rhinitis in RHINE, I-ECRHS and ISAYA, and ratios of ORs between long-term and total baseline participants. There were no significant differences between the ORs of long-term participants and the ORs of all baseline participants in any of the three studies. When stratified by study centers, associations for long-term participants and total baseline participations were more diverse, especially for Pavia, Aarhus and Reykjavik (Additional file 1: e-Table S3 and e-Table S4).

**Table 4:** **Associations between increasing age (5-year intervals) and female sex and the respiratory symptoms wheeze, asthma and rhinitis at baseline (odds ratios with 95**% **confidence intervals) in total baseline population, population at 10-yrs follow-up and population at 20-yrs follow-up, and differences between these groups given as ratios of odds ratios (with bootstrapped 95**% **confidence intervals) in population at 10-yrs follow-up over total baseline population, and in population at 20-yrs follow-up over total baseline population**^**a**^

			***Baseline population OR (95%CI)***	***10-yrs follow-up population OR (95%CI)***	***Ratio of ORs (95%CI)***^***b***^	***20-yrs follow-up population OR (95%CI)***	***Ratio of ORs (95%CI)***^***b***^
Wheeze	Age	RHINE	1.00 (0.98, 1.02)	1.01 (0.98, 1.03)	1.01 (0.99, 1.02)	0.98 (0.95, 1.02)	0.98 (0.96, 1.01)
		Italian ECRHS	**1.10 (1.03, 1.17)**	**1.10 (1.02, 1.19)**	1.00 (0.96, 1.05)	**1.15 (1.01, 1.31)**	1.05 (0.93, 1.18)
		ISAYA	0.97 (0.91, 1.03)	0.96 (0.88, 1.05)	0.99 (0.94, 1.07)	-	-
	Sex	RHINE	0.95 (0.90, 1.02)	0.99 (0.92, 1.07)	1.04 (1.00, 1.08)	0.96 (0.87, 1.05)	1.01 (0.94, 1.07)
		Italian ECRHS	**0.76 (0.64, 0.90)**	**0.74 (0.60, 0.91)**	0.97 (0.86, 1.10)	**0.63 (0.44, 0.91)**	0.83 (0.60, 1.15)
		ISAYA	**0.81 (0.69, 0.96)**	0.79 (0.62, 1.01)	0.98 (0.82, 1.16)	-	-
Asthma	Age	RHINE	0.98 (0.93, 1.02)	0.98 (0.93, 1.03)	1.00 (0.97, 1.03)	0.96 (0.90, 1.02)	0.98 (0.94, 1.03)
		Italian ECRHS	0.99 (0.90, 1.08)	0.94 (0.84, 1.05)	0.95 (0.89, 1.01)	0.88 (0.71, 1.08)	0.89 (0.73, 1.06)
		ISAYA	0.92 (0.83, 1.02)	0.95 (0.83, 1.09)	1.03 (0.93, 1.15)	-	-
	Sex	RHINE	**1.16 (1.02, 1.32)**	**1.21 (1.05, 1.40)**	1.04 (0.97, 1.13)	1.13 (0.95, 1.35)	0.97 (0.87, 1.11)
		Italian ECRHS	0.84 (0.66, 1.08)	0.82 (0.60, 1.12)	0.98 (0.80, 1.18)	0.60 (0.34, 1.06)	0.71 (0.41, 1.18)
		ISAYA	1.08 (0.82, 1.41)	0.94 (0.64, 1.38)	0.87 (0.67, 1.16)	-	-
Rhinitis	Age	RHINE	**0.97 (0.95, 0.99)**	0.97 (0.95, 1.00)	1.00 (0.99, 1.02)	0.97 (0.94, 1.00)	1.00 (0.98, 1.02)
		Italian ECRHS	**0.94 (0.89, 0.99)**	**0.93 (0.88, 0.99)**	0.99 (0.96, 1.03)	0.91 (0.83, 1.01)	0.97 (0.89, 1.07)
		ISAYA	**0.93 (0.88, 0.98)**	**0.91 (0.84, 0.98)**	0.98 (0.93, 1.03)	-	-
	Sex	RHINE	0.99 (0.92, 1.05)	1.01 (0.93, 1.09)	1.02 (0.98, 1.06)	1.01 (0.92, 1.11)	1.02 (0.96, 1.09)
		Italian ECRHS	0.93 (0.81, 1.07)	0.89 (0.75, 1.06)	0.96 (0.86, 1.06)	**0.73 (0.55, 0.97)**	0.78 (0.61, 1.01)
		ISAYA	**0.85 (0.74, 0.99)**	**0.79 (0.64, 0.97)**	0.93 (0.81, 1.06)	-	-

^a^Total baseline population: participants in 1989–92 in RHINE, 1991–93 in Italian ECRHS, 1998–2000 in ISAYA. 10-yrs follow up population: participants in 1999-2000 in RHINE and 1998–2000 in Italian ECRHS, 2008–09 in ISAYA. 20-yrs follow-up population: participants in 2010-12 in RHINE, 2008–09 in Italian ECRHS. ^b^Corrected for inter-dependency between long-term participants and total baseline participants, by using a non-parametric bootstrap method. Significant associations between risk factors and respiratory symptoms are marked in bold font, as well as significant differences between long-term participants and all baseline participants.

### Associations of smoking with respiratory outcomes

In the studies performed in 1998–2000, information on smoking habits was included in RHINE, I-ECRHS and ISAYA. Tables 5, 6 and 7 show ORs for associations of smoking exposure with wheeze, asthma and rhinitis, respectively, as well as the ratios of ORs between 10-yrs follow-up participants and total baseline participants.

**Table 5:** **Associations between smoking status and pack years and wheeze at baseline (odds ratios with 95**% **confidence intervals) in total baseline population and population at 10-yrs follow-up, and differences between these groups given as ratios of odds ratios (with bootstrapped 95**% **confidence intervals) in population at 10-yrs follow-up over total baseline population**^**a**^

		***Baseline population OR (95%CI)***	***10-yrs follow-up population OR (95%CI)***	***Ratio of ORs (95%CI)***^***b***^
Ex-smokers	RHINE	**1.26 (1.13, 1.40)**	**1.26 (1.11, 1.44)**	1.00 (0.93, 1.08)
	Italian ECRHS	1.46 (0.94, 2.27)	1.53 (0.95, 2.48)	1.05 (0.86, 1.28)
	ISAYA	**1.99 (1.53, 2.60)**	**2.11 (1.46, 3.05)**	1.06 (0.81, 1.37)
Current smokers	RHINE	**3.12 (2.84, 3.42)**	**3.21 (2.87, 3.59)**	1.03 (0.97, 1.10)
	Italian ECRHS	**3.31 (2.32, 4.72)**	**3.18 (2.13, 4.75)**	0.96 (0.79, 1.17)
	ISAYA	**3.65 (2.99, 4.45)**	**3.87 (2.92, 5.12)**	1.06 (0.87, 1.31)
1-10 pack years	RHINE	**1.69 (1.53, 1.86)**	**1.71 (1.51, 1.92)**	1.01 (0.94, 1.08)
	Italian ECRHS	**1.58 (1.03, 2.41)**	**1.67 (1.05, 2.67)**	1.06 (0.87, 1.30)
	ISAYA	**2.50 (2.03, 3.08)**	**2.71 (2.02, 3.64)**	1.08 (0.88, 1.33)
10-20 pack years	RHINE	**2.91 (2.57, 3.31)**	**2.92 (2.50, 3.40)**	1.00 (0.92, 1.09)
	Italian ECRHS	**2.77 (1.76, 4.35)**	**2.60 (1.56, 4.33)**	0.94 (0.71, 1.20)
	ISAYA	**3.95 (3.01, 5.19)**	**3.98 (2.66, 5.96)**	1.01 (0.73, 1.36)
20+ pack years	RHINE	**4.37 (3.75, 5.10)**	**3.99 (3.30, 4.82)**	0.91 (0.82, 1.02)
	Italian ECRHS	**4.36 (2.73, 6.98)**	**3.70 (2.16, 6.34)**	0.85 (0.64, 1.10)
	ISAYA	**7.69 (5.49, 10.76)**	**7.89 (4.87, 12.80)**	1.03 (0.71, 1.47)

^a^Total baseline population: participants in 1999-2000 in RHINE and 1998–2000 in Italian ECRHS and ISAYA. 10-yrs follow-up population: participants in 2010–12 in RHINE, 2008–09 in Italian ECRHS and ISAYA. ^b^Corrected for inter-dependency between long-term participants and total baseline participants, by using a non-parametric bootstrap method. Significant associations between risk factors and respiratory symptoms are marked in bold font, as well as significant differences between long-term participants and all baseline participants.

**Table 6:** **Associations between smoking status and pack years and asthma at baseline (odds ratios with 95**% **confidence intervals) in total baseline population and population at 10-yrs follow-up, and differences between these groups given as ratios of odds ratios (with bootstrapped 95**% **confidence intervals) in population at 10-yrs follow-up over total baseline population**^**a**^

		***Baseline population OR (95%CI)***	***10-yrs follow-up population OR (95%CI)***	***Ratio of ORs (95%CI)***^***b***^
Ex-smokers	RHINE	1.05 (0.89, 1.22)	1.09 (0.91, 1.31)	1.04 (0.95, 1.14)
	Italian ECRHS	**1.69 (1.01, 2.83)**	**1.93 (1.11, 3.36)**	1.14 (0.94, 1.42)
	ISAYA	1.35 (0.94, 1.95)	1.47 (0.90, 2.40)	1.09 (0.75, 1.47)
Current smokers	RHINE	1.08 (0.92, 1.25)	1.02 (0.84, 1.24)	0.94 (0.84, 1.07)
	Italian ECRHS	0.72 (0.40, 1.31)	0.67 (0.33, 1.36)	0.93 (0.60, 1.31)
	ISAYA	0.94 (0.68, 1.28)	0.71 (0.44, 1.15)	0.76 (0.51, 1.07)
1-10 pack years	RHINE	1.04 (0.89, 1.21)	1.11 (0.93, 1.34)	1.07 (0.97, 1.18)
	Italian ECRHS	1.35 (0.80, 2.30)	1.59 (0.89, 2.84)	1.18 (0.91, 1.50)
	ISAYA	1.18 (0.88, 1.60)	1.09 (0.72, 1.67)	0.92 (0.67, 1.22)
10-20 pack years	RHINE	1.04 (0.84, 1.30)	1.07 (0.82, 1.40)	1.03 (0.88, 1.18)
	Italian ECRHS	0.77 (0.35, 1.68)	0.72 (0.29, 1.76)	0.94 (0.48, 1.35)
	ISAYA	0.91 (0.56, 1.46)	0.84 (0.41, 1.69)	0.92 (0.49, 1.49)
20+ pack years	RHINE	1.19 (0.91, 1.56)	1.05 (0.74, 1.48)	0.88 (0.69, 1.08)
	Italian ECRHS	0.68 (0.29, 1.61)	0.58 (0.21, 1.57)	0.85 (0.41, 1.26)
	ISAYA	0.71 (0.35, 1.47)	0.45 (0.14, 1.50)	0.63 (0.19, 1.43)

^a^Total baseline population: participants in 1999-2000 in RHINE and 1998–2000 in Italian ECRHS and ISAYA. 10-yrs follow-up population: participants in 2010–12 in RHINE, 2008–09 in Italian ECRHS and ISAYA. ^b^Corrected for inter-dependency between long-term participants and total baseline participants, by using a non-parametric bootstrap method. Significant associations between risk factors and respiratory symptoms are marked in bold font, as well as significant differences between long-term participants and all baseline participants.

**Table 7:** **Associations between smoking status and pack years and rhinitis at baseline (odds ratios with 95**% **confidence intervals) in total baseline population and population at 10-yrs follow-up, and differences between these groups given as ratios of odds ratios (with bootstrapped 95**% **confidence intervals) in population at 10-yrs follow-up over total baseline population**^**a**^

		***Baseline population OR (95%CI)***	***10-yrs follow-up population OR (95%CI)***	***Ratio of ORs (95%CI)***^***b***^
Ex-smokers	RHINE	0.92 (0.83, 1.01)	0.92 (0.83, 1.03)	1.00 (0.95, 1.06)
	Italian ECRHS	0.97 (0.71, 1.32)	0.93 (0.66, 1.32)	0.96 (0.82, 1.12)
	ISAYA	1.10 (0.89, 1.35)	1.03 (0.78, 1.37)	0.94 (0.76, 1.14)
Current smokers	RHINE	**0.80 (0.73, 0.88)**	**0.80 (0.71, 0.89)**	1.00 (0.94, 1.07)
	Italian ECRHS	**0.68 (0.51, 0.92)**	0.71 (0.50, 1.00)	1.04 (0.87, 1.22)
	ISAYA	**0.71 (0.60, 0.85)**	**0.59 (0.46, 0.76)**	**0.83 (0.69, 0.99)**
1-10 pack years	RHINE	0.92 (0.84, 1.01)	0.91 (0.81, 1.01)	0.99 (0.93, 1.04)
	Italian ECRHS	1.00 (0.75, 1.35)	1.06 (0.76, 1.47)	1.06 (0.90, 1.23)
	ISAYA	0.99 (0.84, 1.17)	0.96 (0.76, 1.20)	0.97 (0.83, 1.12)
10-20 pack years	RHINE	**0.77 (0.67, 0.88)**	**0.80 (0.68, 0.94)**	1.04 (0.96, 1.14)
	Italian ECRHS	**0.58 (0.38, 0.89)**	**0.55 (0.34, 0.89)**	0.95 (0.72, 1.18)
	ISAYA	**0.57 (0.43, 0.76)**	**0.32 (0.20, 0.51)**	**0.56 (0.36, 0.79)**
20+ pack years	RHINE	**0.67 (0.56, 0.80)**	**0.69 (0.56, 0.86)**	1.03 (0.91, 1.16)
	Italian ECRHS	**0.63 (0.40, 0.99)**	0.62 (0.37, 1.04)	0.98 (0.75, 1.25)
	ISAYA	**0.41 (0.27, 0.62)**	**0.29 (0.15, 0.56)**	0.71 (0.38, 1.12)

^a^Total baseline population: participants in 1999-2000 in RHINE and 1998–2000 in Italian ECRHS and ISAYA. 10-yrs follow-up population: participants in 2010–12 in RHINE, 2008–09 in Italian ECRHS and ISAYA. ^b^Corrected for inter-dependency between long-term participants and total baseline participants, by using a non-parametric bootstrap method. Significant associations between risk factors and respiratory symptoms are marked in bold font, as well as significant differences between long-term participants and all baseline participants.

There was increased OR for wheeze with smoking in all three studies. The ORs differed slightly between 10-yrs follow-up participants and total baseline participants, but all relative differences were below 15% and not statistically significant (Table 5). There were no significant associations between smoking and asthma in the three studies, with the exception of an association between ex-smokers and asthma in I-ECRHS (Table 6). None of the ORs between long-term participants and total baseline population differed significantly from each other.

Current smoking and smoking more than 10 pack years were both associated with a lower OR for rhinitis in all study centers (Table 7). In RHINE and I-ECRHS there were no differences in ORs between long-term and all baseline participants, while the OR of current smokers and subjects with 10–20 pack years were significantly lower for long-term than all baseline participants in ISAYA (17% and 44% relative difference, respectively).

## Discussion

The present study of long-term participation in RHINE, I-ECRHS and ISAYA showed that increasing age and female sex were predictors for long-term participations. When comparing long-term participants to all baseline participants, we found lower baseline prevalence of several respiratory symptoms among long-term participants compared to all baseline participants. However, analyses of exposure-outcome associations showed only minor differences between long-term participants and all baseline participants.

### Characteristics and bias associated with long-term association

That older people and women are more prone to participate in follow-up studies than younger subjects and men is in line with previous studies [22-27]. Several studies have furthermore shown that non-responders tend to be smokers to a larger degree than responders [22,25-28]. At the baseline studies in RHINE and I-ECRHS, we did not have information on smoking habits among responders and non-responders, but a previous report from ISAYA showed that smokers were over-represented among late responders compared to early responders [15].

Many studies report response rates as an indicator of the data generalizability. However, it has been pointed out that even studies with high response rates may have biased effect estimates if the non-response is not random [7]. Results from the present study indicated that long-term participants had less respiratory symptoms compared to all baseline participants in RHINE, ISAYA and I-ECRHS, with the exception of rhinitis. In the literature, we find studies that are both in accordance and in discordance with our results [14,15,22,25,28-30]. These differences between studies show the importance of assessing selection bias in every longitudinal study, rather than simply stating the response rate [26,31].

Interestingly, two of the reports that are in contradiction with the results from our study are from I-ECRHS and ISAYA [14,15]. These reports showed that there was a higher prevalence of respiratory symptoms among early responders than late responders in baseline ISAYA and I-ECRHS, and a higher symptom prevalence among those who participated in both the screening part and the clinical part of the baseline ECRHS than among those who participated only in the screening part. That the Italian papers have focused on late responders at baseline may partly explain the diverging results regarding symptom prevalence. Although responding late, they were baseline participants, and as such these subjects are included in the total baseline population of the present study. Also, even if those who participated in the screening questionnaire but refused to take part in the clinical part of the baseline ECRHS can be defined as non-responders in the clinical study, the follow-up time between these two parts of the baseline ECRHS was short and consequently not comparable to the present study.

In both RHINE and I-ECRHS we defined 20-yrs follow-up participation as subjects who participated at baseline and *both* follow-up studies. This response rate was 53% in RHINE, but a noteworthy proportion of subjects participated at baseline and at the second follow-up, but not in the first follow-up. If considering participants who were part of the baseline and the second follow-up study, regardless of participation in the first follow-up study, the 20-yrs response rate in RHINE was raised from 53% to 61% (13 128 participants). Additional analyses showed that the tendencies both regarding prevalence estimates and exposure-outcome associations remained unchanged regardless of how we defined 20-yrs follow-up participants (results not shown).

While baseline prevalence estimates were somewhat altered when excluding those lost to follow-up in the present study, the 9 exposure-outcome associations analysed were mainly unchanged. Such a tendency has also been noted by others [16,23,32,33], and may indicate that internal causal associations are less vulnerable to selection bias than prevalence estimates. It should be noted, however, that the focus of the present paper was associations at baseline. Exposure-outcome associations based on one of the follow-up studies with both the follow-up population and those lost to follow-up included might have resulted in different estimates. Since those lost to follow-up per definition will never be included in a follow-up study, this will of course be a purely theoretical speculation.

Future prevalence reports from RHINE, I-ECRHS and ISAYA should take the results from the present study into account and interpret prevalence rates accordingly. For instance, knowing that the baseline prevalence of wheeze in RHINE was 8% lower among long-term participants than among the total baseline participants should have consequences for the interpretation of wheeze prevalence in a later follow-up study. If wheeze prevalence at a follow-up study is for instance 25%, we should take into account that the “true” wheeze prevalence is likely to be approximately 8% higher, i.e. 27%. Also, knowing that the baseline prevalence of rhinitis in ISAYA was 14% higher among long-term participants than among the total baseline participants would infer a similar interpretation of rhinitis prevalence in a later follow-up study: a rhinitis prevalence of for instance 20% in a follow-up study would indicate a “true” rhinitis prevalence to be 14% higher, i.e. 22.8%.

Bias in baseline prevalence estimates may also have consequences for follow-up estimations on incidence, remission and in some instances also risks. The lower baseline prevalence of respiratory symptoms among long-term participants as compared to total baseline participants that we found in the present study may indicate a healthy survivor effect in the study. Such an effect is most commonly observed in association with occupation, in that persons who remain employed tend to be healthier than those who leave employment. However, it is also plausible that persons who continue to participate in a study is healthier than persons who quit their study participation, especially in a study with such a long follow-up period as the RHINE and the Italian ECRHS have. Incidence and remission estimates in the follow-up stages of these studies may both be under-estimated compared to true population estimates if the follow-up population is generally healthier than the total population. However, in the present study we did not find very large variations in baseline prevalence estimates, and the effects on incidence and remission estimates later on in the study are consequently likely to be small. Future incidence investigations based on the three studies covered here should nevertheless take into account the observed baseline differences between total baseline participants and long-term participants in the interpretation of results.

### Merits and limits of the study

The main strengths of this study are 1) the large sample size, 2) the extensive follow-up time, and 3) the use of a methodology that is well suited to assess size and direction of selection bias in long-term follow-up. Certain limitations should also be acknowledged: firstly, the lack of information on predictors for baseline participation. We have examined long-term participation but know little of potential selection bias at baseline. Secondly, the three studies in this report have not been conducted at exactly the same points in time. This is especially relevant for ISAYA, which started 10 years after ECRHS and RHINE. However, since the results are essentially the same between studies with regard to follow-up participation patterns, we do not believe that the time aspect is vital in this context. Thirdly, we have focused on a limited amount of selected exposures and outcomes. To be sure that loss to follow-up does not bias other effect estimates, all possible exposures and outcomes should in principle have been examined in the same way. However, this is not feasible. Although many associations remain to be analysed, we believe that the selection of different exposures and outcomes in the present paper gives an indication of the validity of RHINE, I-ECRHS and ISAYA.

## Conclusions

To conclude, increasing age and female sex were predictors for long-term participation. Prevalence estimates from the follow-up populations should be interpreted with some caution in future reports from RHINE, I-ECRHS and ISAYA since they tended to be slightly lower than for the total baseline population. Exposure-outcome associations, on the other hand, were mainly unchanged by loss to follow-up. Although response rates varied between studies, the present results indicate high validity in the data from RHINE, I-ECRHS and ISAYA.

## Competing interests

The authors declare that they have no competing interests.

## Authors’ contributions

AJ, GV, BB, BF, KF, TG, MH, CJ, RJ, EL, FM, ER, FGR, EWS, VS, TS, TDS, CS, KT, MW and RdM carried out the studies included in the present paper (RHINE, I-ECRHS and ISAYA), participated in study design, coordination and data collection. AJ performed the statistical analyses and drafted the manuscript. GV, RMN and RdM participated with guidance regarding the statistical analyses and helped draft the manuscript. All authors provided input on previous versions of the manuscript, and all authors read and approved the final manuscript.

## Pre-publication history

The pre-publication history for this paper can be accessed here:

http://www.biomedcentral.com/1471-2466/14/63/prepub

## Supplementary Material

Additional file 1 Online supplement material. Click here for file
