# Supplementary material for: Longterm follow-up in European respiratory health studies – patterns and implications
Source: BMC Pulm Med. 2014 Apr 16;14:63. doi: 10.1186/1471-2466-14-63 (PMC4021078; doi:10.1186/1471-2466-14-63)
Supplement: Additional file 1 — Online supplement material. [file 1471-2466-14-63-S1.docx]

**LONGTERM FOLLOW-UP IN EUROPEAN RESPIRATORY HEALTH STUDIES – PATTERNS AND IMPLICATIONS**

ONLINE SUPPLEMENT MATERIAL

SUPPLEMENTAL INFORMATION FOR THE METHODS SECTION, “SELECTED OUTCOMES AND EXPOSURES” PARAGRAPH

Exact wording in the questionnaires for the selected main outcomes wheeze, asthma and rhinitis:

1) Wheeze was defined as an affirmative answer to the question “Have you had wheezing or whistling in your chest at any time in the last 12 months?”

2) Asthma was defined as an affirmative answer to the question “Have you had an attack of asthma in the last 12 months?” and/or affirmative answer to the question “Are you currently taking any medicine (including inhalers, aerosols or tablets) for asthma?”

3) Rhinitis was defined as an affirmative answer to the question “Do you have any nasal allergies including hay fever?”.

SUPPLEMENTAL INFORMATION FOR THE METHODS SECTION, “STUDY POPULATION” PARAGRAPH

The names of Ethics Committees that approved the study:

Aarhus: the Scientific Ethical Committee for Region Midt, Denmark

Bergen: the Regional Ethics Committee in Western Norway

Gothenburg, Umeå and Uppsala: the Regional Ethical Review Board of Uppsala University

Reykjavik: the National Bioethics Committee, Iceland

Tartu: Research Ethics Committee of the University of Tartu

Verona: Comitato Etico per la Sperimentazione dell'Azienda Ospedaliera Istituti Ospitalieri di Verona

Pavia: Comitato di Bioetica della Fondazione IRCCS Policlinico San Matteo di Pavia

Turin: Comitato Etico dell’Azienda Sanitaria Locale TO/2 di Torino

Sassari: Comitato di Bioetica dell’Azienda Sanitaria Locale di Sassari

E-Table S1. Differences between long-term participants and baseline participants in baseline prevalence of asthma, rhinitis and wheeze, across study centers. Differences are given as ratios of baseline symptom prevalence (with bootstrapped 95% confidence intervals) in population at 10-yrs follow-up over total baseline population, and in population at 20-yrs follow-up over total baseline population. ^a^

|  |  | Symptom | STAGE 1+2 ^b^ | STAGE 1+2+3 ^b^ |
| --- | --- | --- | --- | --- |
| RHINE | Aarhus | Wheeze | **0.94 (0.90, 0.98)** | **0.86 (0.81, 0.91)** |
|  |  | Asthma | 0.93 (0.83, 1.04) | **0.82 (0.67, 0.97)** |
|  |  | Rhinitis | 0.98 (0.93, 1.03) | 0.96 (0.89, 1.03) |
|  | Bergen | Wheeze | 0.98 (0.94, 1.02) | **0.93 (0.88, 0.99)** |
|  |  | Asthma | 1.01 (0.90, 1.10) | 0.97 (0.82, 1.12) |
|  |  | Rhinitis | 1.03 (0.99, 1.07) | 1.01 (0.94, 1.08) |
|  | Gothenburg | Wheeze | 0.99 (0.95, 1.03) | **0.93 (0.87, 0.99)** |
|  |  | Asthma | 1.02 (0.93, 1.10) | 0.87 (0.72, 1.01) |
|  |  | Rhinitis | 1.02 (0.99, 1.06) | 0.95 (0.89, 1.02) |
|  | Reykjavik | Wheeze | 0.96 (0.91, 1.02) | 0.94 (0.87, 1.01) |
|  |  | Asthma | **1.20 (1.09, 1.31)** | **1.22 (1.05, 1.40)** |
|  |  | Rhinitis | **1.06 (1.01, 1.11)** | **1.10 (1.03, 1.17)** |
|  | Tartu | Wheeze | 0.96 (0.92, 1.01) | **0.91 (0.83, 0.99)** |
|  |  | Asthma | 0.88 (0.68, 1.08) | 0.94 (0.62, 1.28) |
|  |  | Rhinitis | 1.02 (0.96, 1.07) | **1.11 (1.01, 1.21)** |
|  | Umea | Wheeze | 1.00 (0.96, 1.03) | 1.01 (0.95, 1.07) |
|  |  | Asthma | 1.00 (0.94, 1.06) | 1.06 (0.94, 1.18) |
|  |  | Rhinitis | 1.02 (0.99, 1.05) | **1.08 (1.02, 1.14)** |
|  | Uppsala | Wheeze | 0.98 (0.94, 1.01) | 0.95 (0.88, 1.01) |
|  |  | Asthma | 0.97 (0.89, 1.04) | 0.91 (0.79, 1.04) |
|  |  | Rhinitis | 0.99 (0.96, 1.02) | 1.01 (0.96, 1.07) |
| Italian | Verona | Wheeze | **1.10 (1.02, 1.18)** | 1.05 (0.93, 1.16) |
| ECRHS |  | Asthma | 1.00 (0.86, 1.13) | 0.89 (0.70, 1.06) |
|  |  | Rhinitis | 1.01 (0.95, 1.08) | 1.08 (0.99, 1.17) |
|  | Pavia | Wheeze | 1.00 (0.91, 1.09) | - |
|  |  | Asthma | 0.91 (0.72, 1.08) | - |
|  |  | Rhinitis | 1.04 (0.97, 1.10) | - |
|  | Turin | Wheeze | 0.96 (0.86, 1.05) | - |
|  |  | Asthma | 1.06 (0.91, 1.21) | - |
|  |  | Rhinitis | 0.97 (0.89, 1.05) | - |
| ISAYA | Verona | Wheeze | 0.95 (0.87, 1.03) | - |
|  |  | Asthma | 0.98 (0.84, 1.11) | - |
|  |  | Rhinitis | 0.98 (0.92, 1.04) | - |
|  | Sassari | Wheeze | 0.92 (0.80, 1.04) | - |
|  |  | Asthma | 0.94 (0.73, 1.16) | - |
|  |  | Rhinitis | 1.06 (0.96, 1.17) | - |

^a^ Stage 1 (total baseline population): participants in 1989-92 in RHINE, 1991-93 in Italian ECRHS, 1998-2000 in ISAYA. Stage 2 (10-yrs follow up population): participants in 1999-2000 in RHINE, 1998-2000 in Italian ECRHS, and 2008-09 in ISAYA. Stage 3 (20-yrs follow-up population): participants in 2010-12 in RHINE and 2008-09 in Italian ECRHS.

^b^ Corrected for inter-dependency between long-term participants and total baseline participants, by using a non-parametric bootstrap method. Significant differences between long-term participants and all baseline participants are marked in bold font.

E-Table S2. Prevalence (with 95% CI) of respiratory symptoms at baseline (other symptoms than the 3 listed in the main paper) in total baseline population, population at 10-yrs follow-up and population at 20-yrs follow-up, and differences between these groups given as ratios of baseline symptom prevalence (with bootstrapped 95% CI) in populations at 10-yrs follow-up and at 20-yrs follow-up, both over total baseline population. ^a^

|  |  | Baseline population  (95%CI) | 10-yrs follow-up population (95%CI) | Ratio of prevalences (95%CI) ^b^ | 20-yrs follow-up population (95%CI) | Ratio of prevalences (95%CI) ^b^ |
| --- | --- | --- | --- | --- | --- | --- |
| Wheeze | RHINE | 11.3% (10.9, 11.8) | 10.9% (10.4, 11.4) | **0.96 (0.94, 0.99)** | 10.5% (9.9, 11.1) | **0.93 (0.89, 0.96)** |
| with | Italian ECRHS | 2.7% (2.3, 3.1) | 2.7% (2.2, 3.2) | 1.00 (0.87, 1.11) | 1.4% (0.7, 2.0) | **0.52 (0.30, 0.73)** |
| dyspnea | ISAYA | 3.0% (2.5, 3.5) | 2.4% (1.7, 3.0) | **0.80 (0.63, 0.95)** | - | - |
| Wheeze | RHINE | 13.8% (13.4, 14.3) | 13.4% (12.9, 14.0) | **0.97 (0.95, 0.99)** | 12.7% (12.1, 13.3) | **0.92 (0.89, 0.95)** |
| without | Italian ECRHS | 6.9% (6.2, 7.5) | 6.8% (6.0, 7.5) | 0.99 (0.91, 1.05) | 6.6% (5.3, 8.0) | 0.96 (0.79, 1.13) |
| cold | ISAYA | 10.7% (9.8, 11.6) | 10.0% (8.7, 11.2) | 0.93 (0.85, 1.01) | - | - |
| Waking | RHINE | 11.8% (11.3, 12.3) | 11.5% (11.0, 12.0) | **0.97 (0.95, 1.00)** | 11.1% (10.4, 11.7) | **0.94 (0.90, 0.98)** |
| with chest | Italian ECRHS | 8.1% (7.4, 8.8) | 8.1% (7.2, 9.0) | 1.00 (0.93, 1.06) | 8.2% (6.7, 9.6) | 1.01 (0.85, 1.17) |
| tightness | ISAYA | 10.3% (9.4, 11.2) | 9.2% (8.0, 10.4) | **0.89 (0.82, 0.98)** | - | - |
| Waking | RHINE | 5.5% (5.2, 5.8) | 5.2% (4.9, 5.6) | **0.95 (0.92, 0.99)** | 4.7% (4.3, 5.1) | **0.85 (0.81, 0.92)** |
| with | Italian ECRHS | 7.1% (6.5, 7.8) | 6.9% (6.1, 7.7) | 0.97 (0.90, 1.04) | 5.5% (4.2, 6.7) | **0.77 (0.62, 0.93)** |
| dyspnea | ISAYA | 7.0% (6.2, 7.8) | 6.4% (5.4, 7.4) | 0.91 (0.82, 1.02) | - | - |
| Asthma | RHINE | 3.0% (2.7, 3.2) | 3.0% (2.8, 3.3) | 1.00 (0.98, 1.06) | 2.8% (2.5, 3.1) | 0.93 (0.88, 1.02) |
| attack | Italian ECRHS | 3.8% (3.3, 4.2) | 3.7% (3.1, 4.3) | 0.97 (0.89, 1.09) | 3.3% (2.3, 4.3) | 0.87 (0.66, 1.11) |
|  | ISAYA | 4.3% (3.7, 4.9) | 4.1% (3.3, 5.0) | 0.95 (0.82, 1.10) | - | - |
| Current | RHINE | 3.7% (3.4, 3.9) | 3.7% (3.4, 4.0) | 1.00 (0.98, 1.06) | 3.6% (3.2, 3.9) | 0.97 (0.92, 1.05) |
| asthma | Italian ECRHS | 2.0% (1.7, 2.4) | 2.1% (1.7, 2.6) | 1.05 (0.92, 1.19) | 1.6% (1.0, 2.3) | 0.80 (0.53, 1.15) |
| medication | ISAYA | 3.4% (2.8, 3.9) | 3.3% (2.6, 4.1) | 0.97 (0.83, 1.13) | - | - |

^a^ Total baseline population: participants in 1989-92 in RHINE, 1991-93 in Italian ECRHS, 1998-2000 in ISAYA. 10-yrs follow up population: participants in 1999-2000 in RHINE, 1998-2000 in Italian ECRHS, and 2008-09 in ISAYA. 20-yrs follow-up population: participants in 2010-12 in RHINE and 2008-09 in Italian ECRHS.

^b^ Corrected for inter-dependency between long-term participants and total baseline participants, by using a non-parametric bootstrap method. Significant differences between long-term participants and all baseline participants are marked in bold font.

E-Table S3. Differences in associations between increasing age (5-year intervals) and the respiratory symptoms asthma, rhinitis and wheeze at baseline (odds ratios with 95% confidence intervals) between total baseline population, population at 10-yrs follow-up and population at 20-yrs follow-up. The differences are given as ratios of odds ratios (with bootstrapped 95% confidence intervals) in population at 10-yrs follow-up over total baseline population (1+2), and in population at 20-yrs follow-up over total baseline population (1+2+3). ^a^

| Age | Wheeze | Asthma | Rhinitis |
| --- | --- | --- | --- |
| Aarhus 1+2 | **0.97 (0.93, 1.00)** | 0.98 (0.89, 1.08) | **1.05 (1.01, 1.09)** |
| Aarhus 1+2+3 | **0.92 (0.86, 0.97)** | 0.93 (0.81, 1.07) | **1.07 (1.01, 1.14)** |
| Bergen 1+2 | 1.01 (0.98, 1.05) | 0.99 (0.91, 1.07) | 1.01 (0.98, 1.05) |
| Bergen 1+2+3 | 1.00 (0.94, 1.06) | 1.04 (0.91, 1.19) | 0.99 (0.94, 1.05) |
| Gothenburg 1+2 | 1.01 (0.98, 1.05) | 1.01 (0.95, 1.08) | 0.99 (0.96, 1.03) |
| Gothenburg 1+2+3 | 0.99 (0.93, 1.05) | 0.99 (0.88, 1.12) | 1.01 (0.95, 1.08) |
| Reykjavik 1+2 | 0.99 (0.94, 1.04) | 0.94 (0.87, 1.01) | 0.98 (0.94, 1.02) |
| Reykjavik 1+2+3 | 0.97 (0.91, 1.03) | 0.92 (0.82, 1.03) | 0.97 (0.92, 1.03) |
| Tartu 1+2 | 1.03 (0.99, 1.08) | 1.17 (0.98, 1.43) | 1.02 (0.97, 1.07) |
| Tartu 1+2+3 | 1.00 (0.92, 1.08) | 0.94 (0.68, 1.27) | 0.98 (0.90, 1.06) |
| Umea 1+2 | **1.03 (1.00, 1.07)** | 1.04 (0.99, 1.09) | 1.01 (0.98, 1.04) |
| Umea 1+2+3 | 1.03 (0.97, 1.09) | 1.05 (0.96, 1.15) | 1.01 (0.96, 1.07) |
| Uppsala 1+2 | 1.00 (0.97, 1.03) | 0.97 (0.92, 1.02) | 0.98 (0.96, 1.01) |
| Uppsala 1+2+3 | 1.00 (0.94, 1.05) | 0.93 (0.84, 1.02) | 0.99 (0.94, 1.04) |
| Verona 1+2 | 1.01 (0.94, 1.07) | 0.92 (0.82, 1.01) | 0.97 (0.92, 1.02) |
| Verona 1+2+3 | 1.03 (0.94, 1.13) | 0.93 (0.78, 1.07) | 0.97 (0.90, 1.04) |
| Pavia 1+2 | 1.02 (0.95, 1.11) | **1.18 (1.03, 1.51)** | 0.97 (0.92, 1.02) |
| Turin 1+2 | 0.98 (0.91, 1.06) | 0.92 (0.82, 1.02) | 1.03 (0.96, 1.10) |
| Verona ISAYA 1+2 | 1.01 (0.93, 1.08) | 1.06 (0.95, 1.19) | 0.98 (0.93, 1.04) |
| Sassari ISAYA 1+2 | 1.02 (0.90, 1.14) | 0.96 (0.79, 1.15) | 0.97 (0.88, 1.08) |

^a^ Stage 1 (total baseline population): participants in 1989-92 in RHINE, 1991-93 in Italian ECRHS, 1998-2000 in ISAYA. Stage 2 (10-yrs follow up population): participants in 1999-2000 in RHINE, 1998-2000 in Italian ECRHS, and 2008-09 in ISAYA. Stage 3 (20-yrs follow-up population): participants in 2010-12 in RHINE and 2008-09 in Italian ECRHS.

95% confidence intervals are corrected for inter-dependency between long-term participants and total baseline participants, by using a non-parametric bootstrap method. Significant differences between long-term participants and all baseline participants are marked in bold font.

E-Table S4. Differences in associations between female sex and the respiratory symptoms asthma, rhinitis and wheeze at baseline (odds ratios with 95% confidence intervals) between total baseline population, population at 10-yrs follow-up and population at 20-yrs follow-up. The differences are given as ratios of odds ratios (with bootstrapped 95% confidence intervals) in population at 10-yrs follow-up over total baseline population (1+2), and in population at 20-yrs follow-up over total baseline population (1+2+3). ^a^

| Sex | Wheeze | Asthma | Rhinitis |
| --- | --- | --- | --- |
| Aarhus 1+2 | 1.02 (0.92, 1.13) | **1.34 (1.05, 1.78)** | 0.97 (0.86, 1.09) |
| Aarhus 1+2+3 | 1.00 (0.86, 1.17) | 1.37 (0.93, 2.10) | 0.97 (0.81, 1.16) |
| Bergen 1+2 | 1.02 (0.93, 1.12) | 1.06 (0.86, 1.33) | 1.06 (0.96, 1.17) |
| Bergen 1+2+3 | 1.03 (0.88, 1.20) | 1.04 (0.74, 1.49) | 1.05 (0.90, 1.23) |
| Gothenburg 1+2 | 1.07 (0.96, 1.19) | 1.06 (0.90, 1.28) | 1.07 (0.97, 1.17) |
| Gothenburg 1+2+3 | 1.10 (0.93, 1.34) | 0.86 (0.59, 1.22) | 1.07 (0.89, 1.29) |
| Reykjavik 1+2 | 1.04 (0.91, 1.20) | **0.80 (0.66, 0.97)** | 0.92 (0.82, 1.05) |
| Reykjavik 1+2+3 | 1.01 (0.83, 1.23) | 0.93 (0.68, 1.31) | 0.92 (0.79, 1.09) |
| Tartu 1+2 | 1.01 (0.90, 1.15) | 1.41 (0.88, 2.61) | 1.08 (0.94, 1.23) |
| Tartu 1+2+3 | 0.99 (0.79, 1.23) | 2.11 (0.99, 6.88) | 1.10 (0.88, 1.40) |
| Umea 1+2 | 1.07 (0.98, 1.17) | 1.13 (0.99, 1.30) | 1.08 (0.99, 1.17) |
| Umea 1+2+3 | 0.90 (0.76, 1.06) | 0.85 (0.68, 1.09) | 1.01 (0.87, 1.17) |
| Uppsala 1+2 | 1.01 (0.92, 1.11) | 0.91 (0.77, 1.06) | 0.98 (0.90, 1.07) |
| Uppsala 1+2+3 | 0.96 (0.81, 1.13) | 0.90 (0.68, 1.22) | 1.04 (0.89, 1.19) |
| Verona 1+2 | 0.93 (0.78, 1.11) | 0.85 (0.64, 1.15) | 0.89 (0.76, 1.04) |
| Verona 1+2+3 | 0.92 (0.71, 1.20) | 0.65 (0.41, 1.00) | **0.81 (0.66, 0.99)** |
| Pavia 1+2 | **1.23 (1.05, 1.51)** | 1.16 (0.76, 1.80) | 1.16 (0.99, 1.39) |
| Turin 1+2 | 1.00 (0.79, 1.26) | 1.09 (0.80, 1.49) | 0.93 (0.77, 1.12) |
| Verona ISAYA 1+2 | 0.90 (0.74, 1.09) | 0.94 (0.70, 1.26) | 0.88 (0.75, 1.03) |
| Sassari ISAYA 1+2 | 1.10 (0.81, 1.52) | 0.86 (0.52, 1.45) | 1.09 (0.84, 1.39) |

^a^ Stage 1 (total baseline population): participants in 1989-92 in RHINE, 1991-93 in Italian ECRHS, 1998-2000 in ISAYA. Stage 2 (10-yrs follow up population): participants in 1999-2000 in RHINE, 1998-2000 in Italian ECRHS, and 2008-09 in ISAYA. Stage 3 (20-yrs follow-up population): participants in 2010-12 in RHINE and 2008-09 in Italian ECRHS.

95% confidence intervals are corrected for inter-dependency between long-term participants and total baseline participants, by using a non-parametric bootstrap method. Significant differences between long-term participants and all baseline participants are marked in bold font.
